# The deprescribing rainbow: a conceptual framework highlighting the importance of patient context when stopping medication in older people

**DOI:** 10.1186/s12877-018-0978-x

**Published:** 2018-11-29

**Authors:** Adam Todd, Jesse Jansen, Jim Colvin, Andrew J. McLachlan

**Affiliations:** 1 School of Pharmacy, Faculty of Applied Sciences, Newcastle University, NE17RU, Newcastle upon Tyne, UK; 2 Wiser Healthcare, Sydney School of Public Health, The University of Sydney, Rm 226a, Edward Ford Building A27, Sydney, 2006 NSW Australia; 3 Centre for Medical Psychology and Evidence-based Decision-making (CeMPED), The University of Sydney, Rm 226a, Edward Ford Building A27, Sydney, 2006 NSW Australia; 4 Health Consumers New South Wales, Sydney, Australia; 5 The University of Sydney, Sydney Pharmacy School and Centre for Education and Research on Ageing, The University of Sydney, Sydney, 2006 NSW Australia

**Keywords:** Deprescribing, Polypharmacy, Shared decision making, Person-centered care

## Abstract

The area of “deprescribing” has rapidly expanded in recent years as a positive intervention to reduce inappropriate polypharmacy and improve health outcomes for (older) people with multimorbidity. While our understanding of deprescribing as a process has greatly improved and existing approaches all have patient-centered elements, there is still limited literature exploring the importance of the individual patient context in deprescribing decision-making. This is clearly an important consideration to ensure that any deprescribing approach is ethical, respectful, and successful. To address this gap in the literature, we have developed a conceptual framework in the form of a rainbow – with five different deprescribing determinants – and place the person at the center of the deprescribing process. This framework is informed by literature on patient-centered care for older people and people with multimorbidity. We illustrate the potential application of this framework to a complex patient case to highlight the importance of the different clinical, psychological, social, financial and physical deprescribing determinants, and how this approach could be adopted by those working in clinical practice.

**Electronic supplementary material:**

The online version of this article (10.1186/s12877-018-0978-x) contains supplementary material, which is available to authorized users.

## Background

A clear example of “too much medicine” is inappropriate polypharmacy in older people. Polypharmacy – usually defined as taking 5 or more medications [1] – is very common: recent estimates suggest that around 25 to 40% of adults aged over 65 years are prescribed at least 5 medications [2] – and a significant proportion of such medications are considered “inappropriate” [3]. While older people can benefit from taking multiple medications, inappropriate polypharmacy – where harms outweigh the benefits – can be a significant risk and cost to the individual older patient, as well as society in general. Indeed, inappropriate polypharmacy can lead to the development of adverse drug events, which may cause adverse drug reactions, drug-drug interactions, hospitalization and, in some cases, death [4]. Taken together, inappropriate polypharmacy poses a unique dilemma regarding the balance of benefit and harm, autonomy and justice [5, 6].

As part of the Choosing Wisely movement and efforts to reduce low value care, a large body of literature has emerged focusing on deprescribing [7–9], which has been described as ‘*the process of withdrawal of an inappropriate medication, supervised by a health care professional with the goal of managing polypharmacy and improving outcomes*’ [10]. In view of this, a number of protocols have been developed that help identify inappropriate polypharmacy [11, 12] and consider the process of deprescribing [13, 14]; these protocols seek to support healthcare professionals when making complex decisions about stopping or reducing medications. Although most of these protocols incorporate elements of patient-centered care, a key challenge that has been somewhat overlooked is that the feasibility and effectiveness of deprescribing greatly depends on the individual patient context [15, 16]. For example, deprescribing medication when a patient is nearing the end of life may require a completely different approach when compared to deprescribing medication in an otherwise healthy older adult [17, 18]. It makes intuitive sense to take a patient centered approach to deprescribing, after all the patient is a unique expert on his or her personal circumstances, goals and preferences. It is also supported by studies showing that patient centered care and involving patients in decisions generally has a positive effect on patient–clinician communication as well as patient involvement and patient outcomes, such as knowledge, satisfaction, confidence in the decision, adherence, quality of life and health outcomes [19–24]. Reviews in the context of polypharmacy and deprescribing show positive effects of patient-centered interventions on knowledge, satisfaction, identification of medication related problems [25] and suggest that interventions that require active patient involvement are more effective than interventions that do not [26].

The aim of this paper is to put the person at the center of the deprescribing process, with the underlying assumption that deprescribing will be more successful if it is respectful of the individual patient context and circumstances.

**Fig. 1:**
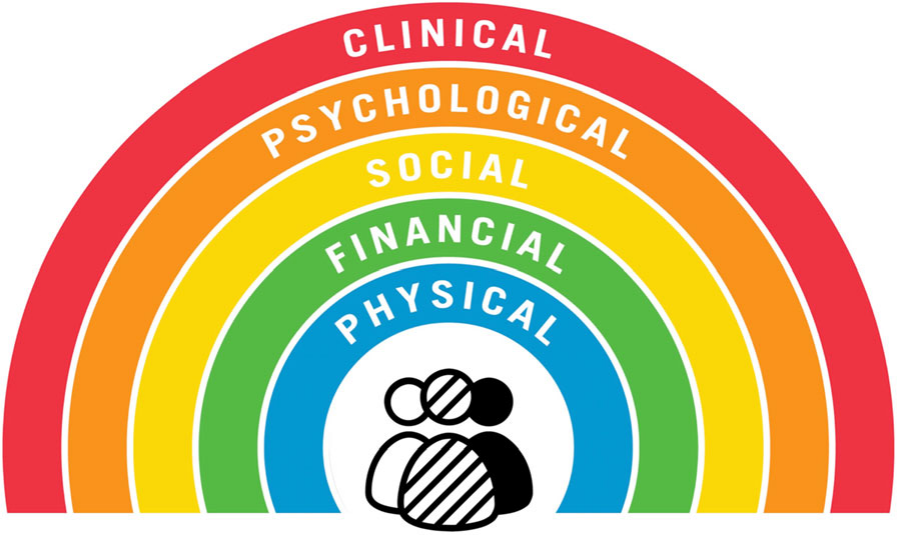
The deprescribing rainbow highlighting the clinical, psychological, social, financial and physical determinants that should be considered when deciding to undertake an episode of deprescribing

Similar to current discussions around prescribing guidelines [27], deprescribing protocols are important as a summary of the best available evidence and an outline of the different deprescribing steps. We argue that additional resources are needed to help clinicians and patients determine if deprescribing is appropriate given their context, and determine how medications can be carefully ceased or doses reduced to achieve the best health goals and outcomes desired by the patient. To achieve our aim, we have developed a conceptual framework in the form of a deprescribing rainbow (Fig. 1), outlining the clinical, psychological, social, financial and physical determinants that should be considered in conjunction with clinical deprescribing guidelines and together with the patient when deciding to undertake an episode of deprescribing to ensure the process has the best chance of success (Table 1). This framework incorporates principles of patient-centered communication and decision-making for people with multimorbidity and the concept of *Minimally Disruptive Medicine* [16, 28–32]. The rainbow analogy highlights the heterogeneity of the older population and how patient-centered deprescribing needs to acknowledge this diversity and individuality [16]. A rainbow, which has been previously used as a model to conceptulise health [33], also symbolizes that deprescribing should be recognized as a positive intervention aimed at improving outcomes important to the patient, and that the relationship between these factors is fluent and may change over time. We illustrate the potential application of the deprescribing rainbow to a hypothetical patient case to highlight the importance of the five deprescribing determinants, as described below. The hypothetical case was initially developed from the clinical experience of authors AT, JJ, AM; it was then discussed informally with healthcare professionals who have responsibility for prescribing medication to older people to ensure the case was realistic and relevant to clinical practice; finally, and importantly, the case was discussed and refined with a patient and carer group, and also with co-author JC, who is a health consumer representative for this work, to ensure it was relevant and meaningful to patients and other healthcare users.

**Table 1:**
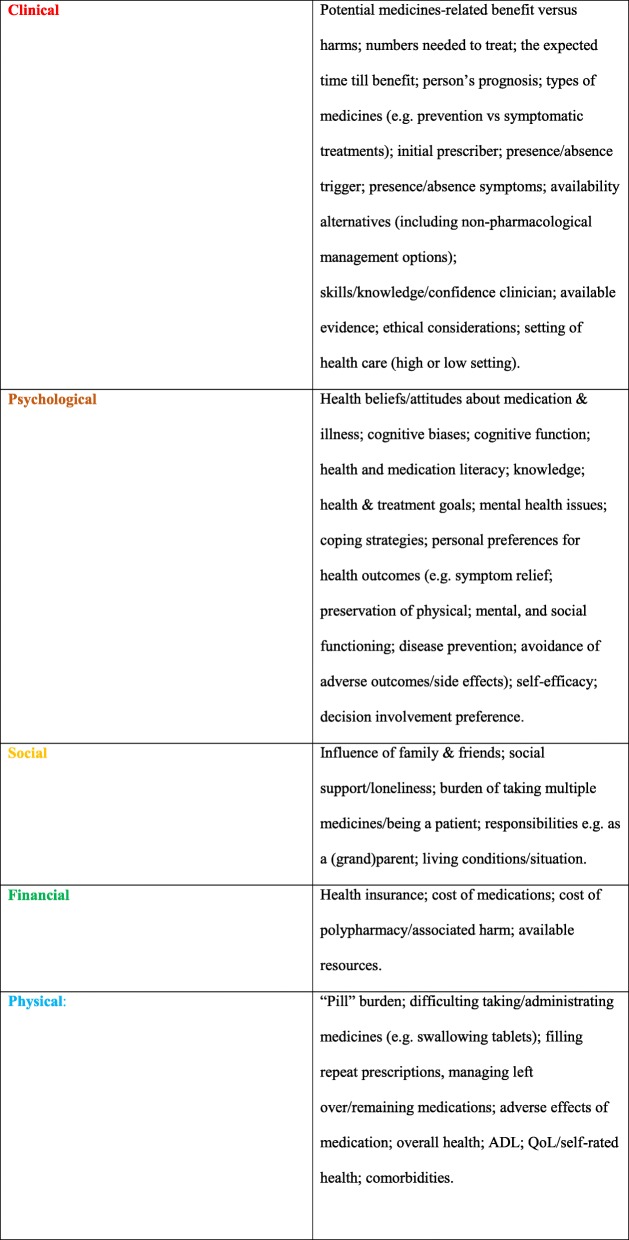
Deprescribing context: examples of clinical, psychological, social, financial and physical factors to take into account^a^

^a^The elements of the deprescribing rainbow are informed by literature on patient-centered  care for older people and people with multimorbidity [16, 28–32]

### A hypothetical case example showing polypharmacy in an older adult

Mrs. EF is a 66-year old female who has recently been diagnosed with stage IV lung cancer, specifically with squamous cell histology; she is currently undergoing chemotherapy treatment with carboplatin and gemcitabine with palliative intent. Based upon her current disease status, her life expectancy is estimated to be approximately 12 months. Her co-morbid health conditions include COPD, hypertension, atrial fibrillation, and depression for which she is prescribed the following medications:

- Salbutamol inhaler ‘1 puff when required’
- Tiotropium inhaler ‘1 puff once daily’
- Bendroflumethiazide 2.5 mg ‘one tablet once daily’
- Lisinopril 20 mg ‘one tablet once daily’
- Atenolol 50 mg ‘one tablet once daily’
- Paracetamol 500 mg ‘two tablets four times daily’
- Morphine sulphate MR 10 mg ‘one tablet twice daily’
- Senna 7.5 mg ‘two tablets at night’
- Citalopram 20 mg ‘one tablet once daily’
- Aspirin 75 mg ‘one tablet once daily’
- Lansoprazole 15 mg ‘one capsule once daily’
- Warfarin 3 mg adjusted per INR (target INR range 2–3)
- Simvastatin 40 mg ‘one tablet at night’
- Calcium 500 mg ‘one tablet twice daily’

EF continues to smoke around 15 cigarettes per day, and has done for the last thirty years; she does not drink any alcohol. She is married, and has two children who are both at University studying Medicine; her husband recently had an ischemic stroke: as such, EF believes it is essential to keep her blood pressure under control, as she, too, is anxious she might have a stroke one day – especially since her cancer diagnosis. In view of this, she has recently purchased a blood pressure machine to monitor her, and her husband’s, blood pressure; she does this at least three times a week, and yesterday her sitting blood pressure was 127 / 75 mmHg, while her standing blood pressure was 107 / 63 mmHg. She is pleased with this result, and believes the medication must be “doing its job”. She tolerates her antihypertensive medications reasonably well, but admits the atenolol makes her feel “tired all the time”.

To keep her cholesterol under control, she previously only ever ate fresh fruit and vegetables, although since her cancer diagnosis, this has become less important to her, as she wants to spend time with her husband when he eats out at restaurants with friends. She acknowledges her cholesterol might increase through this lifestyle change, but is not too worried, as she is also taking her simvastatin.

EF also buys a calcium supplement from the local health food shop; she takes this to keep her bones “strong”. She has used this supplement for a number of years, and started to take it, because after reading in a magazine, she learned that the bisphosphonate medication her doctor previously prescribed for osteoporosis can cause “esophageal cancer”. EF believes that, as calcium is a “natural mineral”, it is not really a drug in the true sense of the word, and is 100 % “safe”.

Out of all of her medication, EF ranks taking the warfarin as the most important: this is because her eldest son, a fourth-year medical student, who has recently spent time in an INR clinic on placement, has explained that warfarin is a “blood thinner” and prevents clots from forming. Even though she believes the warfarin gives her terrible constipation, she has promised her son that she will continue to take it. This is in contrast to the aspirin, which she also understands is a blood thinner, but it is not as “strong” as her warfarin; as the aspirin gives her indigestion, she often misses doses, but is comfortable with this knowing the warfarin will still “do its job”.

She manages her medication quite well, although acknowledges that she does not have the energy to drive to the doctor’s surgery anymore to order her repeat medication prescriptions. Nowadays, because of her lung cancer, she relies on the local pharmacy to deliver her medications; this can also be challenging, as, in recent times, she frequently misses the delivery driver, as she is often at the hospital attending various medical outpatient appointments.

Even though EF does not really like taking medication, and has difficultly in swallowing some of her tablets, she explains that, as she has paid into a medical insurance plan all of her life, she wants something back from her policy, and even if her medications only give her the smallest benefit, she still wants to continue to take them.

## Discussion

The hypothetical case of Mrs. EF is clearly complex: her medication record and medical history highlight complex multimorbidity and examples of inappropriate polypharmacy [34], and Mrs. EF would likely benefit from deprescribing some of her medicines. We consider the hypothetical case of EF in the context of the deprescribing rainbow, as described below. We describe a process of identifying which clinical, psychological, social, financial and physical determinants are a priority for a patient.

The rainbow is intented to be used in conjunction with clinical deprescribing frameworks (see Additional file 1 in which the elements of the rainbow are discussed in relation to the well-cited deprescribing framework by Scott and colleagues [14]). Example questions that can be used to support the translation of the deprescribing rainbow into clinical practice are shown in Additional file 2. These questions are informed by literature on patient centered communication and patient centered deprescribing. Deprescribing is a time consuming (often ongoing) process, involving many different healthcare professionals. A comprehensive and interdisciplinary approach is required to ensure that deprescribing is patient centered and takes into account the complex patient context. Similar to, for example, a comprehensive geriatric assessment, this could take place over multiple consultations and could involve different types of healthcare professionals, depending on the context.

### Clinical determinants

The patient has stage IV lung cancer with diminished life expectancy; EF also has a number of comorbid health conditions, including COPD, hypertension, atrial fibrillation, and depression. Her last blood pressure reading was 127 / 75 mmHg (standing) and 107 / 63 mmHg (sitting), suggesting that EF has postural hypotension. This increases the risk of EF having a fall – and the risk of harm from a fall is further compounded because Mrs. EF is receiving the anticoagulant warfarin. The patient has reported a number of adverse effects that are likely from (or worsened by) her current medications: atenolol causes fatigue, morphine causes constipation, while the aspirin causes dyspepsia. Owing to these adverse effects, EF regularly misses doses of the aspirin, although remains compliant with atenolol and morphine. Mrs. EF has also experienced some prescribing cascades: lansoprazole to treat epigastric adverse effects of aspirin, senna to treat the constipation adverse effects of morphine [35]. Based on even the best evidence, the number needed to treat and the time to benefit for simvastatin [36] and calcium [37] suggest there is a level of futility in their use in Mrs. EF given her life expectancy. Mrs. EF is also taking a number of medicines that are considered “high risk” in vulnerable older people, including diuretics, opioid analgesics and anticoagulants [38]. From a clinical standpoint alone, the following preventative medications have questionable clinical benefit in this patient given her prognosis, and could be considered for deprescribing: lisinopril, bendroflumethaizide, atenolol, aspirin, warfarin, simvastatin, and calcium [34].

### Psychological determinants

Due to her anxieties about having a stroke, the patient believes it is vitally important to have low blood pressure; this is linked to her husband’s previous history of ischemic stroke. Mrs. EF has a CHA_2_DS_2_-VASc Score for Atrial Fibrillation Stroke Risk of 2 (or higher given her history of cancer) suggesting she is in the moderate-high risk range of ischemic stroke. While Mrs. EF believes it is important to have low blood pressure, the clinical reality is that it could cause her more harm than benefit owing to the increased risk of having a fall. However, it is well known that both clinician and patients tend to overestimate the benefits of medicines and underestimate the harms [39, 40]. The literature suggests that an individualised approach is warrented for EF [41] – with a consensus guideline suggesting a target systolic blood pressure of 160–190 mmHg would be adequate [42]. Before there is any discussion or consideration of stopping or reducing the antihypertensive medication, her misguided beliefs about hypertension should be addressed. The fact that EF measures her blood pressure at home is also important context to the case, as reducing or stopping the anti-hypertensive medication may cause her blood pressure to increase, which may cause her substantial anxiety. If these issues are not acknowledged and overcome, it is likely that any attempt to stop or reduce the antihypertensive medication would be unsuccessful. It is also possible that, despite having a discussion about hypertension, EF might not be ready – from a psychological perspective – to reduce or stop her medication; it is important in this context that the prescriber is supportive of EF’s decision. Failure to acknowledge this psychological determinant, may have a negative influence on EF regarding any future deprescribing decisions.

The next psychological factor relates to EF’s wish to spend more time with her husband by eating out with friends. Her previous anxieties around having a poor diet have reduced, although taking the simvastatin appears important to EF in this regard, and may provide some positive reinforcement to eating out with her husband. While trial evidence suggests that stopping statins in this patient group is safe [43, 44], discontinuing this medication may cause her anxiety to return, which in turn, may act as a barrier to her eating out, and thus spending time with her husband. It should be explained to EF that, although the statin will reduce her cholesterol, it is unlikely that this will give her any survival benefit; she should be encouraged to eat out with her husband, and that it is perfectly safe to do so, without the need for the statin therapy. In contrast to her blood pressure, EF is unlikely to be in a situation where she can routinely monitor her cholesterol; this context may help promote effective deprescribing of the statin.

The final point for the psychological determinants relates to the calcium, which is being used by the patient to prevent osteoporosis. EF believes that because the calcium is a natural supplement, it is free from harm. This belief is further strengthened in that she purchases the calcium from a health food shop, rather than a pharmacy. Evidence from the literature suggests that the effectiveness of calcium therapy for osteoporosis is limited [37]; while the calcium is unlikely to cause significant harm, EF is also using a thiazide diuretic and has malignant disease, which could increase the possibility of developing hypercalcemia. Any discussion around stopping the calcium should address the issue that all medications – whether purchased over the counter or prescribed by their physician – can have harmful effects.

### Social determinants

An important consideration in relation to the social determinants of deprescribing is associated with the warfarin and aspirin, and how this relates to EF’s family – in particular, the eldest son, a fourth-year medical student. EF has made a promise to her son that she will continue to take the warfarin which provides important context to the case. Considerations for stopping warfarin without involving the son in the decision-making process may have negative consequences for their relationship. The fact that EF has recently spent time in an INR clinic adds further emphasis to this point. Previous literature has shown that, in the context of life limiting illness, caregivers or family members act as gatekeepers of care when it comes to managing medication [45]. In view of this any discussion about stopping the warfarin therapy should involve EF’s son. Such discussions should focus on the risks versus benefits of warfarin therapy. It is also important to explain that stopping the warfarin therapy is not a sign that EF is being given up on: it should be considered as a positive intervention, and emphasised that EF will be carefully monitored and put back on warfarin if needed. If EF or her sons are not yet accepting of her terminal illness and reduced life expectancy (previous studies refer to this acceptance as a “transition” [45]), a possible compromise may be to stop the aspirin therapy in the first instance. Given that EF is not adherent to her aspirin therapy, and this medication does not appear to be as important to EF or her family, this could be a reasonable compromise. It is it is likely that deprescribing decisions that do not consider these factors and don’t involve EFs sons would be unsuccessful, and crucially, it may also compromise future deprescribing episodes where the context and circumstances of EF have changed by undermining trust in the clinician.

### Financial determinants

There are two financial determinants pertinent to this case: the first is that EF purchases the calcium supplements from a health food shop. The fact that she is purchasing it herself should be acknowledged and discussed, it may give her a sense of control over her condition; and as we describe above may also provide positive reinforcement with EF’s belief that the calcium is ‘natural’ and ‘safe’. A possible strategy to stop the calcium could explain that there is no difference between prescribed versus over the counter calcium supplements. The second point relating to the financial determinants is that EF has paid for medical insurance all of her life, and feels as though she wants something back from her insurance – possibly by continuing to be prescribed some of her medications. This belief should also be acknowledged and discussed prior to the initiation of any deprescribing episode. It is important to highlight to EF that deprescribing is a positive intervention, which seeks to improve her care. Ultimately, the decision (or not) to undertake an episode of deprescribing should be patient driven, and should never be about simply reducing costs for the healthcare service.

### Physical determinants

The physical determinants in this case relate to EF’s ability to take her medication. At present, she is finding it challenging to swallow some of her medications, and this is unlikely to improve given her disease trajectory. Although people involved in the process of deprescribing tend to focus on the clinical reasons for stopping medication, it is likely that, in this case, the physical challenges of taking multiple medications is having a significant negative impact on the patient’s quality of life. The second physical determent relates to medication management: EF no longer has the energy to drive and order her repeat prescriptions. In view of this, she relies on the pharmacy to deliver her medications, but, as she is often at hospital attending outpatient medical appointments, she frequently misses the delivery driver. In addition to this, the atenolol may also be causing fatigue in EF, which appears to be further compounding her tiredness. These physical determinants should be acknowledged and discussed with EF: in this case, they could be used to help initiate and promote episodes of deprescribing. The atenolol is a good illustration of how all of the different deprescribing determinants should be considered as a continuum in order to achieve a successful episode of deprescribing for just one of medication: the clinical (medication has questionable benefit), the psychological (beliefs about hypertension), the social (relationship with her family), the financial (feels entitled to prescribed medication), and the physical (atenolol may be contributing to fatigue, and overall pill burden).

## Conclusion

We have developed a conceptual framework to promote effective deprescribing in the form of a rainbow, with each segment of the rainbow representing a different deprescribing determinant: clinical, psychological, social, financial and physical. We believe that using the proposed framework alongside existing deprescribing protocols has the potential to support healthcare professionals in their decision-making by highlighting the importance of individual patient context within deprescribing decisions. It is an initial step in an iterative process in which the ideas proposed will be further developed by application, investigation and refinement.

## Additional files

Additional file 1: The relationship between the elements of the deprescribing rainbow and the deprescribing framework as proposed by Scott et al. when applied to the hypothetical patient scenario. (1) key deprescribing step, (2) details deprescribing step (3) elements deprescribing rainbow, (4) elements in Mrs. EF scenario, (5) example response to scenario. (DOCX 31 kb)

Additional file 2: Example key questions to ask yourself (as a healthcare professional) and your patients to address the determinants of the deprescribing rainbow. (1) determinant from the deprescribing rainbow, (2) detailed description determinant, (3) example questions to ask yourself and your patients. (DOCX 23 kb)

## Abbreviations

COPD: (chronic obstructive pulmonary disease)
INR: (international normalized ratio)
MR: (modified release)
