# Supplementary material for: The deprescribing rainbow: a conceptual framework highlighting the importance of patient context when stopping medication in older people
Source: BMC Geriatr. 2018 Nov 29;18:295. doi: 10.1186/s12877-018-0978-x (PMC6267905; doi:10.1186/s12877-018-0978-x)
Supplement: Supplementary file 1 — The relationship between the elements of the deprescribing rainbow and the deprescribing framework as proposed by Scott et al. when applied to the hypothetical patient scenario. (1) key deprescribing step, (2) details deprescribing step (3) elements deprescribing rainbow, (4) elements in Mrs. EF scenario, (5) example response to scenario. (DOCX 31 kb) [file 12877_2018_978_MOESM1_ESM.docx]

**APPENDIX 1. The relationship between the elements of the deprescribing rainbow and the deprescribing framework as proposed by Scott *et al** when applied to the hypothetical patient scenario**

| Key deprescribing step* | Details deprescribing step* | Elements deprescribing rainbow** | Elements in Mrs EF scenario | Example response to scenario (preventative meds only) |
| --- | --- | --- | --- | --- |
| 1. Ascertain all medication the patient is currently taking and the reasons for each one | 1. Ask patient to bring all medication, and medication delivery aids 2. Ask patient about medication not being taken and if so why not (e.g., too expensive, adverse effects) | Clinical: use of alternatives for prescribed medicines (e.g. over the counter, complementary)  Psychological: medication knowledge & understanding; non-adherence difficult to discuss. Cognitive problems, psychological reasons non-adherence.  Social: role carer/others in meds management  Financial: cost as reason for non -adherence  Physical: physical reasons non-adherence | Clinical: use of calcium from the health shop  Psychological: medicine knowledge based on what children told her, misperception conventional medicines ‘natural’ medicines  Social: important role children with medical background  Financial: paying for insurance, wants to get something back from her insurance (paid for an insurance policy all of her life)  Physical: difficulty swallowing some of the medication | - Bendroflumethiazide – hypertension - Lisinopril – hypertension - Aspirin – primary prevention of stroke - Warfarin – atrial fibrillation - Simvastatin – hypercholesterolemia - Calcium – prevention of osteoporosis |
| 2. Consider overall risk of medication-induced harm in determining the required intensity of deprescribing intervention | Ascertain risk according to:  • Medication factors: number of medication, “high-risk” medication, toxicity  • Patient factors: age >80 y, cognitive impairment, multiple comorbidities, substance abuse, multiple prescribers, nonadherence | Clinical: clinical factors to help determine benefit/harm trade off  Social: role carer/others in meds management important in assessing risk and benefits  Psychological: Cognitive impairment, cognitive bias towards benefits over harms  Financial: cost as an important nonclinical harm of polypharmacy and certain expensive medication  Physical: important in this step (e.g. comorbidities, age) | Clinical: stage IV lung cancer, life expectancy approximately 12 months, on chemo, on 12 meds  Social: pressure son to take warfarin  Psychological: wants to avoid a stroke at all cost  Financial: paying for insurance  Physical: comorbidities, side effects aspirin, smoker | - Warfarin (1) is the greatest risk in this patient - Aspirin (2); NSAID in older patient, increase risk of a bleed - Lisinopril (3); increased risk of a fall/can cause renal impairment - Bendroflumethiazide (4); less likely to cause fall; electrolyte imbalance - Simvastatin (5) - Calcium (6) |
| 3. Assess each medication for its eligibility to be stopped:  • No valid indication  • Part of a prescribing cascade  • Actual or potential harm clearly outweighs benefit  • Disease and/or symptom control medication is ineffective, or symptoms have completely resolved  • Preventive medication is unlikely to have any patient-important benefit over remaining lifespan  • Medication is imposing unacceptable treatment burden | • Identify medication prescribed for a diagnosis that is not confirmed; highly atypical presentations; for a confirmed diagnosis but with little evidence of efficacy; or that have no additional benefit after a certain period of continuous use or after a certain age  • Identify medication prescribed to counteract adverse effects of other medication.  • Identify "medication to avoid" in older patients  • Identify medication contraindicated for patient  • Identify medication causing well-known adverse effects  • Ask patient, "Since you started this medicine, has it made such a difference to how you feel that you would prefer to stay on t?" and consider discontinuing the drug if the response is no or probably not  • Ask, "Are you still experiencing any troublesome symptoms? Do you feel the medicine is still required?" Consider discontinuing if the target condition is self-limiting, mild, intermittent, or amenable to nondrug interventions.  • Estimate patient's life expectancy using risk prediction tools or "surprise" question  •Determine patient's expectations and preferences  • Identify drugs unlikely to confer benefit over the patient's remaining lifespan  •Ask the patient, "Apart from side effects, are there any other concerns you have with your medicines?  • Identify drugs that are particularly burdensome (e.g, difficulty swallowing large tablets, out-of-pocket expense, monitoring requirements (such as warfarin sodium) | Clinical: symptomatic vs preventive medicines, making B/H trade off with patient is challenging and requires (risk) communication skills and confidence  Psychological: clinical vs perceived indication meds, bias towards benefits, bias towards status quo, limited understanding (in particular difference symptomatic vs preventive medicines)  Social: influence others on perceived importance meds, support also determines treatment burden  Financial: paying tax, health insurance, cost needs to be considered  Physical: making a benefit: harm trade of is challenging and will depend on e.g. overall health | Clinical: limited benefit preventive  medicines given life expectations  Psychological: BP under control, confirms usefulness meds; statins allow her to enjoy the foods she wants to eat without worrying, Warfarin perceived as essential as it keeps her from having as stroke. Believe that natural medicines is better related to fear of cancer (but prefers to take statins over eating healthily)  Social: promised son to take Warfarin, being social and eat what she wants in restaurants is important  Financial: paying tax  Physical: BP under control | This patient would only consider stopping the below medications, as described, if it was clear that she would benefit. i.e. it was not a cost saving exercise  Hypertension medications (bendroflumethiazide/lisinopril) are a ‘no go’ in this patient in terms of deprescribing; she wants to take them, due to the fact her husband has had a stroke. Even if the benefits versus risks are explained to her, she is adamant she wants to take them. This belief is reinforced the BP monitoring; if it were to increase this would cause the patient anxiety  Would think about stopping the calcium. Patient believes the calcium is not a ‘real drug’, so it’s not as important as the other medication. She buys it too, so stopping it will save her money  Would also consider stopping the simvastatin providing it was clear she could still go and eat out with her husband; education around this issue is important  Stopping the warfarin would be a ‘no go’. The patient is not bothered about taking it – unlike the antihypertensive medication (given it also causes constipation) but feels she will let her son down if it is stopped; she believes her son will think she has ‘given up’. She makes it clear though that she doesn’t think she gets any benefit from it taking it.  Would happily stop the aspirin – she doesn’t even know why it has been prescribed, given she takes the warfarin too. |
| 4. Prioritize drugs for discontinuation | Deciding order of discontinuation using criteria:  (1) those with the greatest harm and least benefit;  (2) those easiest to stop, i.e, lowest likelihood of withdrawal reactions or disease rebound;  (3) those that the patient is most willing to stop first  Suggested approach is to rank drugs from high harm/low benefit to low harm/high benefit and discontinue the former in sequential order. | Clinical: initial prescriber, presence/absence symptoms, availability alternatives. skills/knowledge/confidence clinician in determining prioritization and integrating patient context with clinical information.  Psychological: discrepancy between clinical prioritization and patient prioritization based on psychological, financial and social factors rather than clinical factors. E.g. related to patient goals and preferences and also see cognitive biases above.  Social: willingness to deprescribe is influenced by others, importance of support  Financial: health insurance, cost needs to be taken into account in trade off (e.g. prioritize stopping expensive medicines, cost of alternatives)  Physical: plays a role in determining B/H trade off, repeat prescriptions, left over medication | Clinical:  Psychological: wants to avoid stroke (warfarin) cancer (calcium) and side effects (aspirin), important misconceptions about medicines in particular preventative medicines, misinformed preferences, limited awareness prognosis?  Social: keeping son happy (warfarin) and eating out (statins) are a priority  Financial: sense of entitlement (paying tax)  Physical: | In the case, the patient would rank them in the following order in terms of deprescribing:  (1) aspirin  (2) calcium  (3) simvastatin  (4) antihypertensive medications  (5) warfarin  Ranking the drugs of high harm/low benefit to low harm/high benefit, we would have a different list (warfarin, for example, would be in the top 2 drugs to stop) – the context of the situation is influencing outcome |
| 5. Implement and monitor drug  discontinuation regimen | 1. Explain & agree w patient on management plan 2. Stop 1 drug at a time so that harms and benefits can be attributed to specific medication 3. Wean patients off drugs more likely to cause adverse withdrawal effects 4. Instruct patient/carer on what to look for, report and do in the event of adverse effects occurring 5. Communicate plan to all health professionals and other relevant parties (carers, family) 6. Document the reasons for, and outcomes of, deprescribing | Clinical: involvement of other other prescribers  Psychological: cognitive impairment, limited knowledge may influence adherence to recommendations and reporting/action on withdrawal reactions.  Social: take into account e.g. social support and living arrangement in developing the management plan. Assess the potential burden of management plan.  Financial: ‘wasting’ prescribed medicines, cost of alternatives  Physical: burden of management plan, repeat prescriptions, left over medication | Clinical:  Psychological: increased worry might be an adverse effect of withdrawing some of the medicines  Social: involvement children essential for success management plan  Financial:  Physical: |  |

* The steps described in column 1 and 2 are based on Scott IA, Hilmer SN, Reeve E, et al. Reducing inappropriate polypharmacy: the process of deprescribing. JAMA Intern Med*.* 2015;175(5):827-34.

* *The elements of the deprescribing rainbow are informed by the following summaries of the literature:

- Jansen J, Naganathan V, Carter SM, *et al*. Too much medicine in older people? Deprescribing through shared decision making. BMJ. 2016;353:i2893;
- Muth C, van den Akker M, Blom JW, *et al*. The Ariadne principles: how to handle multimorbidity in primary care consultations. BMC Med. 2014;12:223;
- Patient-centered care for older adults with multiple chronic conditions: a stepwise approach from the American Geriatrics Society: American Geriatrics Society Expert Panel on the Care of Older Adults with Multimorbidity. J Am Geriatr Soc. 2012;60(10):1957-68;
- Leppin AL, Montori VM, Gionfriddo MR. Minimally Disruptive Medicine: A Pragmatically Comprehensive Model for Delivering Care to Patients with Multiple Chronic Conditions. Healthcare (Basel). 2015;3(1):50-63.
- Reeve E, Shakib S, Hendrix I, Roberts MS, Wiese MD. Review of deprescribing processes and development of an evidence‐based, patient‐centred deprescribing process. Br J Clin Pharmacol. 2014;78(4):738-47.
