# Supplementary material for: The deprescribing rainbow: a conceptual framework highlighting the importance of patient context when stopping medication in older people
Source: BMC Geriatr. 2018 Nov 29;18:295. doi: 10.1186/s12877-018-0978-x (PMC6267905; doi:10.1186/s12877-018-0978-x)
Supplement: Supplementary file 2 — Example key questions to ask yourself (as a healthcare professional) and your patients to address the determinants of the deprescribing rainbow. (1) determinant from the deprescribing rainbow, (2) detailed description determinant, (3) example questions to ask yourself and your patients. (DOCX 23 kb) [file 12877_2018_978_MOESM2_ESM.docx]

**Appendix 2. Example key questions to ask yourself (as a healthcare professional)* and your patients to address the determinants of the deprescribing rainbow**

| ***Determinant*** | ***Description*** | ***Example questions***** |
| --- | --- | --- |
| **Clinical** | Potential medicines-related benefit versus harms; numbers needed to treat; the expected time till benefit; person’s prognosis; types of medicines (e.g. prevention vs symptomatic treatments); initial prescriber; presence/absence trigger; presence/absence symptoms; availability alternatives (including non-pharmacological management options); skills/knowledge/confidence clinician; available evidence; ethical considerations; setting of health care (high or low setting). | To ask yourself:  *Are there any medications that are important for the patient to take for a clinical benefit?*  *Are there any medications that the patient is taking that are potentially harmful?*  To ask your patient:  *Are there any medications that are really important to you? And if so, why are they important?*  *Since you started this medication, has it made such a difference to how you feel that you would prefer to stay on it?*  *Are you experiencing any troublesome symptom from your medications?* |
| **Psychological** | Health beliefs/attitudes about medication & illness; cognitive biases; cognitive function; health and medication literacy; knowledge; health & treatment goals; mental health issues; coping strategies; personal preferences for health outcomes (e.g. symptom relief; preservation of physical; mental, and social functioning; disease prevention; avoidance of adverse outcomes/side effects); self-efficacy; decision involvement preference. | To ask yourself:  *What are the patient’s beliefs and understanding about the aims, benefits and harms of their medications?*  *Has the patient previously asked about reducing or stopping medications?*  To ask your patient:  *What are the main reason(s) for taking your medicines?*  *What are you hoping to achieve with your medicines?*  *Are there any concerns you have with your medicines?* |
| **Social** | Influence of family & friends; social support/loneliness; burden of taking multiple medicines/being a patient; responsibilities e.g. as a (grand)parent; living conditions/situation. | To ask yourself:  *What is the patient’s social context, are there any other partners that need to be involved in the deprescribing process?*  *Who is the gatekeeper of care regarding the patient’s medications?*  To ask your patient:  *How do your family feel about your medicines?*  *Where do you find the most joy in your life?* |
| **Financial** | Health insurance; cost of medications; cost of polypharmacy/associated harm; available resources. | To ask yourself:  *Are there any financial aspects that influence the patient taking medications?*  *Are there any financial elements that might influence future care?*  To ask your patient:  *How do you feel about the money you spend on your medications?*  *Are you worried about the financial cost of your care?* |
| **Physical** | “Pill” burden; filling repeat prescriptions, medication management; managing left over/remaining medications; adverse effects of medication; overall health; ADL; QoL/self-rated health; comorbidities. | To ask yourself:  *Does this patient have a high pill burden?*  *Are there any physical barriers that prevent the patient from using their medication?*  To ask your patient:  *Are there any medications that are a burden to you? And if so, why are they burdensome?*  *Are you experiencing any troublesome symptom from your medications?* |

* These questions are intended to prompt healthcare professionals to consider the patient’s context and to ask patients what matters to them

**Questions taken from/informed by the following sources:

- Hashim MJ. Patient-Centered Communication: Basic Skills. Am Fam Physician. 2017;95(1):29-34.
- The ICAN tool. Knowledge & Evaluation Research Unit Mayo Clinic. Available at: <https://kerunit.files.wordpress.com/2015/08/ican-round-3-prototypes-black-and-white-versions-ker3.pdf>
- Patient-centered Communication In Cancer Care: Promoting Healing and Reducing Suffering. National Cancer Institute. Available at: <https://healthcaredelivery.cancer.gov/pcc/pcc_monograph.pdf?file=/pcc/communication/pcc_monograph.pdf>
- Scott IA, Hilmer SN, Reeve E, et al. Reducing inappropriate polypharmacy: the process of deprescribing. JAMA Intern Med*.* 2015;175(5):827-34.
- Wolters M, van Hulten R, Blom L, Bouvy ML. Exploring the concept of patient centred communication for the pharmacy practice. Int J Clin Pharm. 2017;39(6):1145-1156.
